# Fabrication of Superhydrophobic Porous Brass by Chemical Dealloying for Efficient Emulsion Separation

**DOI:** 10.3390/molecules28186509

**Published:** 2023-09-08

**Authors:** Yanbiao Zhou, Qingqing Ye, Yongjun Han, Guoxu He, Changdong Chen

**Affiliations:** School of Chemistry and Environmental Engineering, Pingdingshan University, Pingdingshan 467000, China2738@pdsu.edu.cn (G.H.)

**Keywords:** dealloying, porous brass, Cu_2_O, superhydrophobic, stability

## Abstract

By taking advantage of typical dealloying and subsequent aging methods, a novel homogeneous porous brass with a micro/nano hierarchical structure was prepared without any chemical modification. The treatment of commercial brass with hot concentrated HCl solution caused preferential etching of Zn from Cu_62_Zn_38_ alloy foil, leaving a microporous skeleton with an average tortuous channel size of 1.6 μm for liquid transfer. After storage in the atmosphere for 7 days, the wettability of the dealloyed brass changed from superhydrophilic to superhydrophobic with a contact angle > 156° and sliding angle < 7°. The aging treatment enhanced the hydrophobicity of the brass by the formation of Cu_2_O on the surface. By virtue of the opposite wettability to water and oil, the aged brass separated surfactant-stabilized water-in-oil emulsions with separation efficiency of over 99.4% and permeate flux of about 851 L·m^−2^·h^−1^ even after recycling for 60 times. After 10 times of tape peeling or sandpaper abrasion, the aged brass maintained its superhydrophobicity, indicating its excellent mechanical stability. Moreover, the aged brass still retained its superhydrophobicity after exposure to high temperatures or corrosive solutions, displaying high resistance to extreme environments. The reason may be that the bicontinuous porous structure throughout the whole foil endows stable mechanical properties to tolerate extreme environments. This method should have a promising future in expanding the applications of alloys.

## 1. Introduction

Confronted with the increasing oily wastewater produced from daily life and industrial processes, the removal and recovery of oil stains from water have become a serious global problem [1,2]. Traditional oil/water separation techniques typically suffer from the disadvantages of low separation efficiency, high energy consumption, and sophisticated operation processes. With the development of interface theory, superwetting technology is considered an effective and inexpensive approach for the separation of oily wastewater. The proper porous materials with opposite superwettability toward water and oil, such as superhydrophobic/superoleophilic, superoleophilic/underoil superhydrophobic, and superhydrophilic/underwater superoleophobic properties, have been manufactured and utilized for effective oil/water separation [3]. However, constructing highly uniform and compact porous structures with opposite wettability to oil and water is still an enormous challenge.

The design and development of novel porous metal materials with superwettability have drawn intensive attention to the separation of oily sewage. “Coating” and “etching” are two effective strategies to fabricate porous membranes with opposite wettability. By “coating”, a hierarchically rough film covers or grows out of porous hosts to obtain superwettable membranes [4]. This strategy mainly includes physical coating [5], surface redox [6], and chemical modification [7]. These coated membranes can efficiently purify oily wastewater. However, physical surface coating by polymer is unstable for long-term operation, and surface grafting is too complex for practical application, especially for separating water-in-oil emulsions [8]. The coating of inorganics usually has no robust adhesion to the host and can peel off when subjected to heat or external forces, which is mainly due to the relatively weak interfacial bonding between the inorganics and the host [9]. Therefore, more efforts are required to develop facile methods for preparing membranes with robust structural stability.

Conversely, the “etching” strategy has the potential to avoid the above problems. By “etching”, etching specific components and areas can yield stable micro/nanostructures to some extent [4]. For example, the laser irradiation technique is utilized to etch specific areas for fabricating a microscale vertical pore structure [10]. Chemical etching [11], electrochemical etching [12], and plasma etching [13] methods usually employ corrosive media or plasma to unselectively etch the surface of materials, resulting in the formation of hierarchical rough structures on the surface. As far as we know, only ununiform throughout microporous aluminum sheets with intrinsic superwettability have been fabricated via monolithic chemical etching and electrochemical etching [14,15].

Dealloying, as one of the etching methods, is the process of selective dissolution of more active components from homogeneous alloy, which has become one of the robust and generic approaches for constructing homogeneous porous structures with high surface area [16]. The dealloying method has been successfully applied to produce various porous materials in alloys, such as CuZn [17,18,19,20], CuAl [21], CuFe [22], AuSi [23], LiSn [24], etc. These uniform bicontinuous nanopore structures exhibit unusual properties such as tunable porous size, high permeability, and high structural stability, which render them separation materials with great potential for purifying oily wastewater. For example, Jie et al. demonstrated a combining dealloying and heating approach to achieve a superhydrophobic surface on brass [25]. Li et al. prepared a superhydrophobic copper substrate through a dealloying and subsequent coating method [26]. Although the dealloying technique without surface coating has been utilized to acquire the superhydrophobicity on alloy surfaces [26,27], the development of the superhydrophobic membrane only by dealloying for oil/water separation has not been reported previously.

**Scheme 1:**
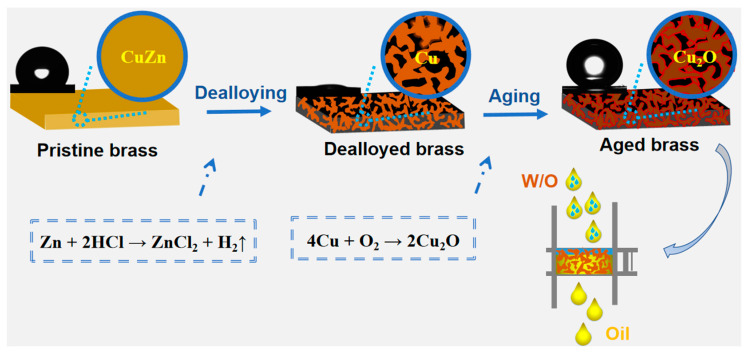
Schematic diagram of dealloying and aging of CuZn alloy foil to prepare a superhydrophobic porous brass for water-in-oil emulsion separation.

Herein, a facile chemical dealloying and subsequent aging process was first developed to produce a highly uniform and compact microporous brass for water-in-oil emulsion separation (Scheme 1). The dissolution of zinc from a commercial bimetallic Cu_62_Zn_38_ alloy brass foil by HCl solution resulted in a bicontinuous structure of well-interconnected pores with a diameter of about 1.6 μm. The bicontinuous Cu framework can act as a channel for liquid transport. After aging for 7 days, the brass changed from superhydrophilic to superhydrophobic. Such a superhydrophobic brass could separate surfactant-stabilized water-in-oil emulsions with a high efficiency of over 99.4% and flux of about 851 L·m^−2^·h^−1^. Even after 60 separation cycles, both separation efficiency and flux remained stable, demonstrating the excellent reusability of the porous brass. Moreover, the as-prepared porous brass exhibited exceptional mechanical, thermal, and chemical stability. This work may provide a new way to construct controllable structures for oil/water separation materials.

## 2. Results and Discussion

### 2.1. Construction of Bicontinuous Porous Structure by Dealloying

**Figure 1:**
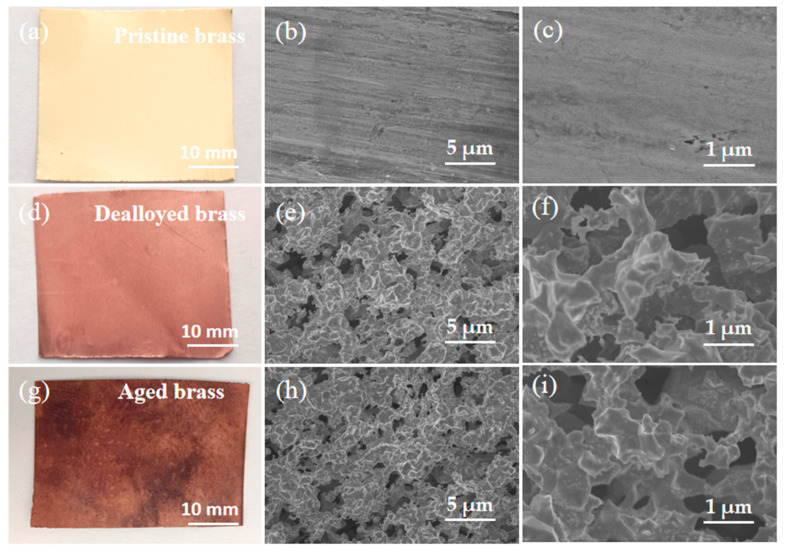
Top-view color photos and scanning electron microscope (SEM) images of samples in different processing stages: (**a**–**c**) pristine brass; (**d**–**f**) dealloyed brass; and (**g**–**i**) aged brass.

The open bicontinuous porous structure was developed by selectively monolithic etching of brass foil (Cu_62_Zn_38_). Typically, chemical dealloying of the commercially available CuZn alloy foil was conducted in a hot concentrated HCl solution. During the dealloying process, the surface color of the CuZn alloy gradually changed from golden–yellow (Figure 1a) to reddish–brown, in accordance with Cu color (Figure 1d). The high concentration and temperature of HCl solution help to form deep and large pores [28]. In the macroscopic view, the Zn phase had been removed from the CuZn alloy, and the indissolvable Cu phase was preserved.

Zn + 2HCl → ZnCl_2_ + H_2_ ↑ (1)

Upon taking out from the hydrochloric acid solution, the dealloyed brass gradually turned dark brown as the aging time increased (Figure 1g). This clearly shows that the dealloying and aging processes can cause a dramatic change in the color of the brass.

Microstructural changes in samples in each processing stage were observed by scanning electron microscope (SEM). The pristine brass foil presented a relatively flat and solid surface (Figure 1b,c). During etching, hydrochloric acid etching proceeded from both surfaces of the brass foil to dissolve Zn. Meanwhile, the copper elements diffused, congregated, and formed interconnected skeletons. At 80 °C, corrosion perforation of the brass sheet occurred after 5 h. Because only a small percentage of pores perforated throughout the brass, the flux was too small to effectively transport liquids. After 8 h, the brass completely transformed to bicontinuous porous copper with irregular micro/nanopores. The diameter of these pores was smaller than 4 μm observed in the structure (Figure 1e,f). Such small micropores are difficult to obtain based on frequently used commercial brass or copper mesh [29,30]. After aging, no obvious difference was detected in the SEM images between the aged and dealloyed brass (Figure 1h,i). The widespread pores in size of the aged brass resulted in the formation of micro/nanoscale hierarchical structures, which played an essential role in the generation of superwettability.

**Figure 2:**
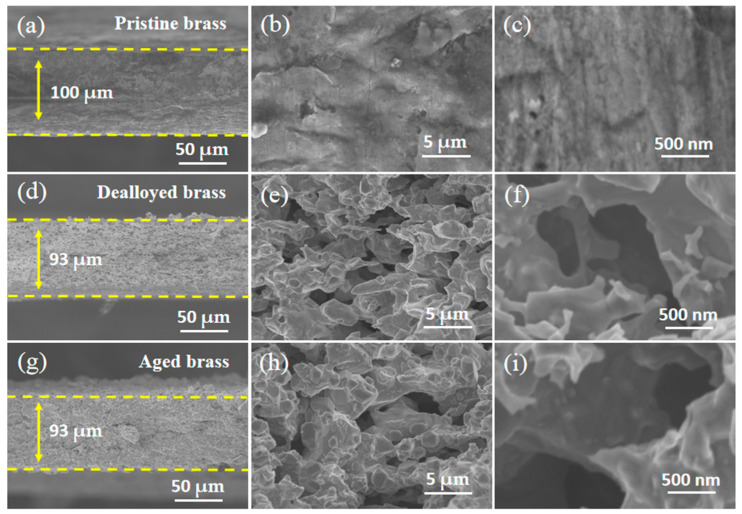
Side-view FESEM images of samples in different processing stages: (**a**–**c**) pristine brass; (**d**–**f**) dealloyed brass; and (**g**–**i**) aged brass. The distance between yellow dotted lines represents sample thicknesses.

Dealloying treatment began on two sides of the CuZn sheet to remove Zn and progressed until corrosion perforation of the brass sheet occurred after 8 h. The cross-section morphology of the dealloyed and aged foil observed by SEM exhibited a highly interconnected porous structure and hierarchical skeleton throughout the whole foil (Figure 2e,f). These micro/nanopores can provide tortuous channels to transport liquids. It is worth noting that the total thickness of the foil after dealloying slightly decreased by about 7% from 100 to 93 μm due to a small amount of dissolution of copper (Figure 2a,d), whereas the thickness and morphology of the foil after aging did not change (Figure 2h,i). To probe the porosity of the dealloyed and aged samples [31], relative bulk density was measured by weighing the mass of the material with a predefined volume to provide insight into the porosity. Compared with that of pristine brass density (8.40 g/cm^3^), the relative density values of the dealloyed and aged brass were around 53.23% and 52.28%, respectively. This means that the porosity of aged brass is slightly lower than that of dealloyed brass, which may be attributed to the partial oxidation of surface Cu.

**Figure 3:**
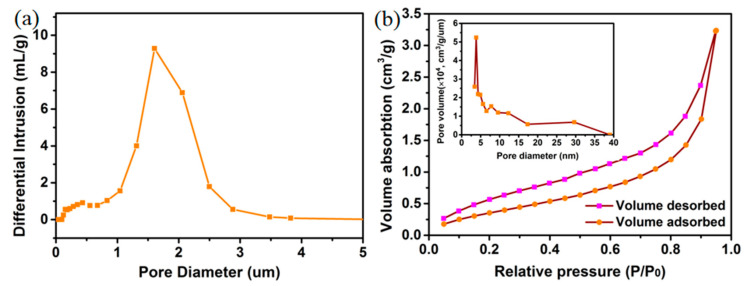
Pore size distribution of the aged brass: (**a**) macroscopic pore size distribution obtained from mercury-intrusion porosimetry measurement; (**b**) N_2_ adsorption/desorption isotherm and corresponding nanopore size distribution.

To further investigate the macroporous distribution of the aged brass, the interconnected pore size distribution was measured by a mercury injection test. The result showed that the porosity of the aged brass was 59.6%, and the pore size ranged from 1.0 to 4.0 µm, with an average of 1.6 μm (Figure 3a), verifying that the aged brass had a compact structure and narrow pore size distribution. The porosity of the aged brass is larger than that obtained by the above gravimetric method, which could be because a small number of pores in the brass might collapse under the pressure required to inject mercury into the micropores [32]. To evaluate the mesoporous and microporous structures, N_2_ adsorption/desorption behaviors were analyzed for the aged brass (Figure 3b). The aged brass presented a surface area of 2.1 m^2^/g and a Brunauer–Emmett–Teller pore size distribution of 2–40 nm (inset in Figure 3b). According to the above SEM observations and pore size distribution, it can be concluded that the aged brass holds a hierarchical architecture with micro/nanoscale pores. All these pores were interconnected to result in unimpeded channels for liquid transport.

### 2.2. Effect of Aging on Chemical Composition

**Figure 4:**
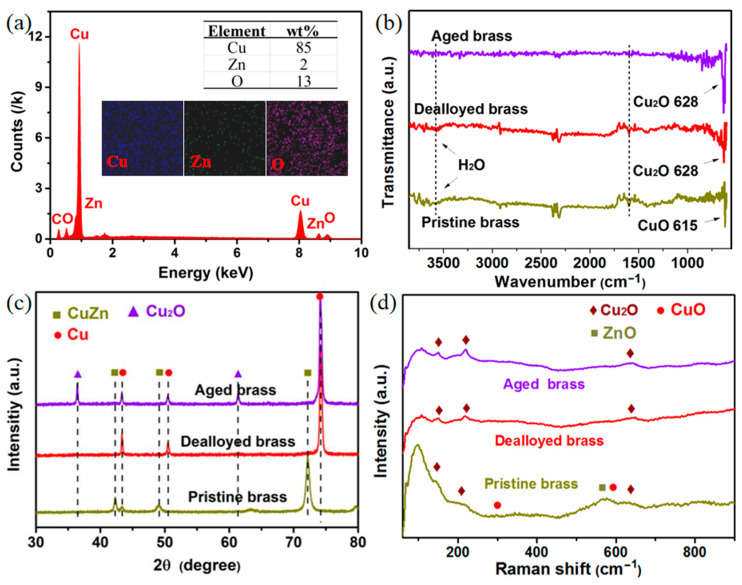
Surface chemistry compositions of samples in different processing stages: (**a**) energy-dispersive X-ray spectrometer (EDS) spectrum analysis and mapping images of the aged brass surface; (**b**) attenuated total reflection Fourier-transform infrared spectra (ATR-FTIR); (**c**) X-ray diffractometer (XRD) patterns; and (**d**) Raman spectra of the pristine, dealloyed and aged brass surface.

The energy-dispersive X-ray spectrometer (EDS) analysis showed that the surface of the pristine brass contained nearly 28 wt% Zn and 59 wt% Cu (Figure 4a). The Zn and Cu elements were evenly distributed in the alloy (Figure S1a), which was very important to constructing a uniform and compact porous structure. After dealloying for 8 h, the zinc content dropped to 2%, while the relative content of Cu increased to 89% (Figure S1b). The element mapping images presented that the Zn in the pristine alloy had been almost completely removed, and porous Cu was retained. The oxygen content of the aged brass was much higher than that of the dealloyed brass but lower than that of the pristine brass, which was consistent with the increase in the relative density for the aged brass, indicating the possible formation of Cu_2_O on the remained skeleton (Figure 4a). However, the Cu/O atomic ratio of the superhydrophobic surface was lower than that of Cu_2_O. The reason is that the EDS measurement penetrates into a depth of a few micrometers into the subsurface and, thus, the detected % of Cu covers both the oxide layer and the copper metal.

The chemical constituents and chemical bonds of the pristine, dealloyed, and aged brass surface were examined by attenuated total reflection Fourier-transform infrared spectra (ATR-FTIR) (Figure 4b). The two series of adsorption peaks at about 3633 and 1602 cm^−1^ are attributed to the presence of adsorbed water [33,34]. This result indicates the existence of a hydration layer on the pristine and dealloyed surface. Upon dealloying, the new adsorption peak for Cu_2_O at 628 cm^−1^ emerged, which implied that the dealloyed brass was coated with Cu_2_O. After the aging treatment, the intensity of the peak at 628 cm^−1^ for Cu_2_O was obviously enhanced, while the absorption bands at 3600 and 1602 cm^−1^ for absorbed water declined, suggesting that the hydrophobic Cu_2_O reduced water absorption.

The crystalline phase of pristine, dealloyed, and aged brass was determined with powder X-ray diffractometer (XRD) patterns (Figure 4c). As expected, the peaks of the pristine brass at 42.3°, 49.2°, and 72.2° matched well with the diffraction peaks of Cu_62_Zn_38_ (JCPDS, 50-1333), which consisted of a solid solution and intermetallic compound composed of Zn and Cu elements, respectively. After dealloying, the characteristic peaks for Cu_62_Zn_38_ disappeared while the three diffraction peaks at around 43.4°, 50.5°, and 74.1° for the indices (111), (200), and (220) crystal planes of pure Cu phase (JCPDS, 04-0836) evidently intensified, suggesting the almost complete dissolution of Zn element from the pristine CuZn alloy and the remaining of almost pure porous Cu [35,36]. After aging for 7 days, diffraction peaks of the Cu phase remained sharp and strong, and two new peaks at 36.4° and 61.4° assigned to (111) and (220) planes of Cu_2_O (JCPDS, 65-3288) appeared. This indicated that a thin passive Cu_2_O layer formed on the porous Cu ligaments [37,38]. Raman spectra were measured to further determine the composition change in the samples at different process stages (Figure 4d). After dealloying, the characteristic peaks of the Cu_2_O phase at 148, 219, and 632 cm^−1^ demonstrated the formation of Cu_2_O on the dealloyed brass. After aging, the intensity of peak respecting Cu_2_O increased significantly, demonstrating that more copper was oxidized over time to cuprous oxide.

**Figure 5:**
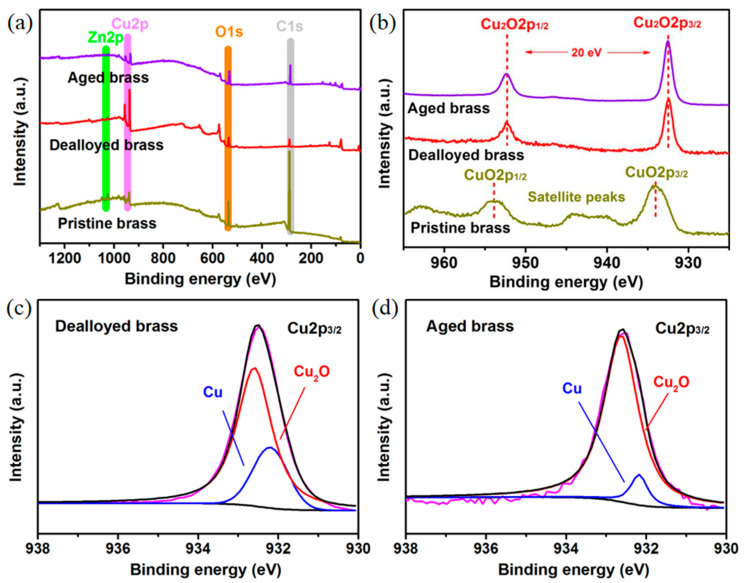
X-ray photoelectron spectroscopy (XPS) spectra of samples in different processing stages: (**a**) wide-spectrum survey; (**b**) high-resolution spectra of Cu2p region; (**c**) deconvolution of Cu2p3/2 of the dealloyed brass; and (**d**) deconvolution of Cu2p3/2 of the aged brass surface.

The X-ray photoelectron spectroscopy (XPS) analysis was utilized to further determine the chemical states of elements on the sample surface (Figure 5). All the binding energies in the XPS analysis were calibrated with C1s at 284.8 eV as the reference. Organic matter might have been adsorbed on the surface from the ambient air [39]. After the dealloying process, the peak intensities of Zn2p3 at 1021.5 eV and Zn2p1 at 1044.5 eV were dramatically diminished (Figure 5a). This confirmed that almost all Zn was selectively leached out from the alloy, and only a very low percentage of Zn was retained inside the matrix. Cu2p spectra were used to determine the valence state of the Cu element. Two sharp Cu2p peaks of the dealloyed brass at 932.4 and 952.4 eV corresponded to Cu2p3/2 and Cu2p1/2, binding the energy of Cu_2_O or Cu (Figure 5b) [40,41]. The LMM Anger spectra could distinguish them with 568 and 570 eV for Cu and Cu_2_O, respectively [42]. On the dealloyed and aged brass surfaces, the Cu LMM peak at 570 eV verified the presence of Cu_2_O. Aging led to an increase in the intensity of the peak for Cu_2_O with time of air exposure, indicating that more copper on the surface of the dealloyed brass gradually oxidized to Cu_2_O (Figure S2). The binding energy of the Cu_2_O phase generally has a slightly lower binding energy than the signal of metallic Cu [43,44]. The high-resolution spectra of Cu2p3/2 of the dealloyed and aged brass could be fitted into two peaks at 932.2, assigned to Cu_2_O, and 932.6 eV, assigned to Cu [45,46]. After aging, the intensity of the Cu peak weakened, while the intensity of the Cu_2_O peak strengthened, indicating that the exterior of the structure gradually oxidized over time (Figure 5c,d) [43]. In general, the combination of EDS, ATR-FTIR, XRD, Raman, and XPS analysis results has proved that more Cu on the dealloyed surface oxidized to Cu_2_O over time.

### 2.3. Effect of Aging Time on Wettability Transition

**Figure 6:**
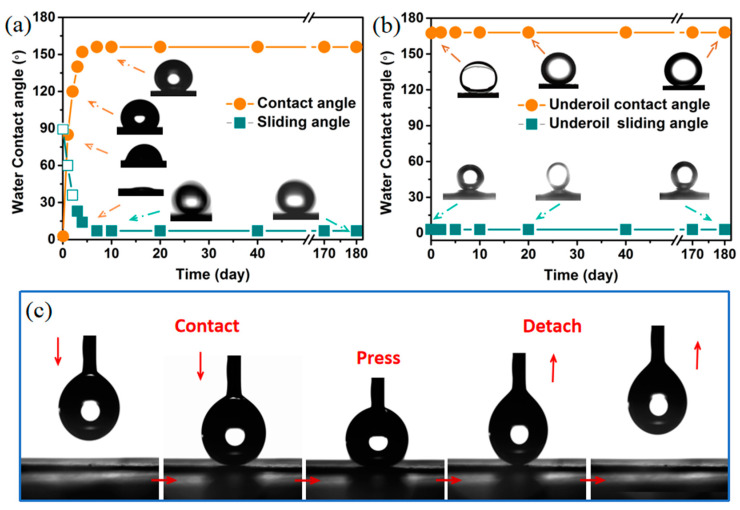
Effect of aging time on surface wettability transition: variation of water CA and SA (**a**) under air. The hollow square represents that an accurate water SA value for water cannot be obtained. (**b**) under oil with aging time; and (**c**) dynamic process of antiwater adhesion in air.

The freshly dealloyed brass surface exhibited a superhydrophilic behavior in the air with water CA close to 0°. However, when the dealloyed brass was exposed to ambient air, the water CA exhibited a sharp increase during the first 5 days, which then slowed to a gradual growth before finally reaching a steady state (Figure 6a). Prolonging the aging time to 7 days, the water CA increased to 156° and SA decreased to 7°, respectively, indicating that the aged surface possessed good superhydrophobicity. On the contrary, oil droplets could swiftly wet and penetrate through the aged brass (Figure S3). Furthermore, when the brass was continually stored for more than 6 months under ambient conditions, the surface could maintain opposite superwettability toward oil and water (Figure 6a). Compared with some previous reports, the aged brass showed better long-term storage stability in ambient atmosphere [47,48,49]. Unlike in the air, the freshly dealloyed brass exhibited superhydrophobicity in oil (kerosene) and did not change with increasing exposure time (Figure 6b). Such long-term storage stability is crucial for the practicality of superhydrophobic surfaces. Moreover, the dynamic repellency toward water was evaluated by squeezing, contacting, pressing, and lifting water to the aged brass surface. No obvious adhesion and droplet deformation were detected as the water droplets lifted from the aged brass surface, indicating the low adhesion of the aged surface (Figure 6c). The excellent water repellency had a synergistic effect on the hierarchical roughness induced by dealloying and the low surface energy introduced by the formation of the Cu_2_O surface. This non-sticking characteristic of the superhydrophobic surface corresponds to the Cassie–Baxter state.

**Figure 7:**
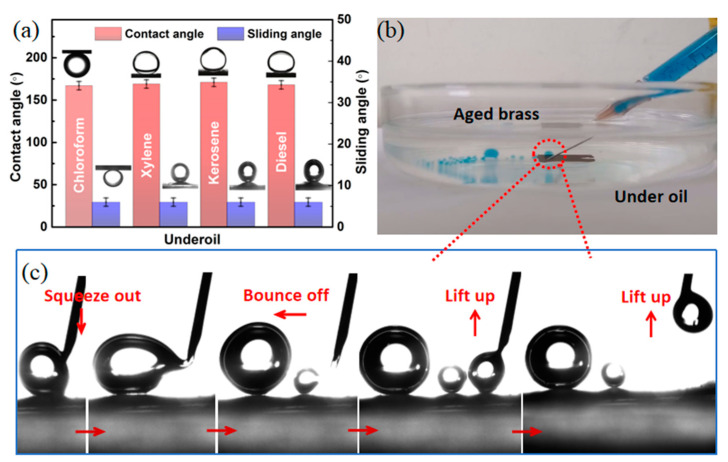
Wetting behavior of the aged brass in oils: (**a**) water CAs and SAs in different types of oils; (**b**,**c**) process of continuous water jet flow under oil.

The wettability of the aged brass surface showed underoil superhydrophobicity after being immersed in different oils (Figure 7a). When immersing the aged brass in oil, oil could be trapped into the hierarchical structure to form oil shielding, thereby reducing the contact area between the solid and water. This was due to the higher adhesion work of oil than that of water. The aged brass presented excellent and stable underoil superhydrophobic properties with a water contact angle (CA) greater than 167° and sliding angle (SA) smaller than 3° in various oils, including chloroform, xylene, kerosene, and diesel, indicating the ultralow adhesion between the water droplet and the brass surface. Furthermore, the dynamic process of a stream of droplets bouncing on the aged brass surface was recorded by a digital camera (Figure 7b) and contact angle meter (Figure 7c), respectively. When a stream of water droplets in kerosene was squeezed from a syringe toward the aged brass, water droplets spontaneously bounced off over the horizontal surface with no water droplets left, indicating that the aged brass surface had an outstanding repellent property for water. After that, when the water was lifted from the aged brass surface, no droplet deformation was detected, further suggesting the low adhesion of the aged surface.

### 2.4. Mechanism of Wetting Transition

Surface chemistry and surface topography are two critical factors affecting surface-wetting properties. HCl etching resulted in the dissolution of Zn, and more internal pure Cu atoms were exposed to the ambient air. Cu is a hydrophilic material in nature [50], and the hydrophilicity increases with roughness. According to Wenzel’s theory, the introduction of hierarchical roughness can amplify the wettability from hydrophilicity to more hydrophilicity. So, after dealloying, the sample exhibited superhydrophilic behavior. However, when exposed to ambient conditions, the dealloyed porous brass immediately reacted with O_2_ from the air to form a solid Cu_2_O surface. The Cu_2_O was generated through the interfacial reaction between Cu and O_2_ by the proposed reaction [51]:

4Cu + O_2_ → 2Cu_2_O (2)

Aging led to the gradual conversion of the upmost layer of hydrophilic Cu to hydrophobic Cu_2_O. Similarly, the hierarchical roughness amplified the hydrophobicity of Cu_2_O into superhydrophobicity according to Wenzel’s theory. Along with the reaction, the hydrophobic surface became more hydrophobic. After being exposed to ambient air for 7 days, the superhydrophilic brass surface reached a superhydrophobic state. This observation was only attributed to the change in surface chemical composition with time, as there was no change in surface morphology during aging (Figure 2).

Three mechanisms have been proposed by others, such as the decomposition of carbon dioxide into carbon with active magnetite [52], adsorption of hydrophobic organic matter from the atmosphere [53,54,55], and creation of hydrophobic groups [56] to explain the wettability transition from superhydrophilicity to hydrophobicity. In our case, there is no energy accountable for the creation of an active magnetite to catalyze the decomposition of carbon dioxide into carbon. Meanwhile, XPS analyses were performed on the dealloyed brass during the aging in the air to evaluate organic adsorption on the surface. When organic matter is adsorbed onto the surface, carbon atoms will be detected. The C1s spectra of the organic matters can be resolved into three peaks with binding energies of about 284.7 eV, 286.4 eV, and 288.6 eV, which are ascribed to the C-C (H), C-O-C, and O-C=O species, respectively [57]. These three species may come respectively from the alkyl, ether, and carboxyl/ester matter spontaneously adsorbed from the air. Among the C-C (H), C-O-C, and O-C=O species, C-C (H) is nonpolar and serves as a hydrophobic region, while the C-O-C and O-C=O species are polar and serve as a hydrophilic region on the surface [54]. It can be seen that the percentage content of the hydrophobic C-C (H) decreased from 76.4% for the dealloyed brass to 39.5% for the aged brass in the total C1s peak area, while the percentage content of the hydrophilic C-O-C and O-C=O species significantly increased from 23.4% to 60.5% (Figure S4). The result indicates that the percentage content of the hydrophilic species increases with aging time. This cannot explain why the aged surface exhibits higher hydrophobicity. Above all, the formation of a very thin passivation layer of hydrophobic Cu_2_O is the reason for the transition from superhyrophilic to superhydrophobic.

**Figure 8:**
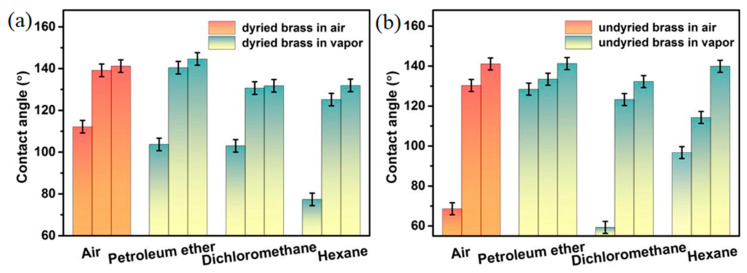
Water CAs of (**a**) dried freshly dealloyed brass and (**b**) undried freshly dealloyed brass after different days of exposure to air.

To further support the mechanism behind wettability switching, dried brass and undried brass, after dealloying, were exposed to four types of the atmosphere, including ambient air, petroleum ether, dichloromethane, and n-hexane. For this purpose, eight freshly dealloyed samples were first rinsed thoroughly in distilled water, and then half of them were dried with cold air from a hair dryer. After that, these dried and undried samples were immediately stored in ambient air or vapors produced by three organic compounds in closed glass bottles for 3 days. Every 24 h, the samples were taken out from each container to measure their water CAs. Following the measurement, these samples were immediately returned back to their containers. The results showed that the water CAs increased as the storage time increased. After three days, the water CAs of both dried and undried samples exceeded 131° (Figure 8). Therein, the water CAs of the samples exposed to three organic compound vapors were no more than that of the corresponding sample in the air. The reason may be that the oxygen concentration in three organic vapors was lower than that in the air, resulting in the aged brass having a lower degree of oxidation in the vapors than that in the air. This result supports the transition mechanism that the variation in surface chemical composition substantially induces an increase in hydrophobicity.

### 2.5. Water-in-Oil Emulsion Separation with High Efficiency

**Figure 9:**
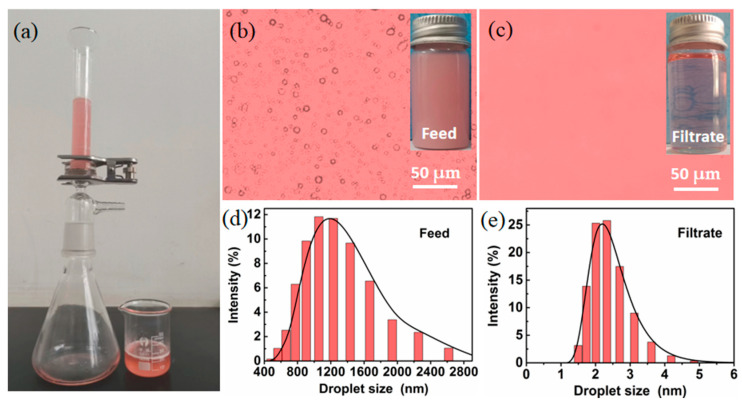
Separation performance of surfactant-stabilized water-in-chloroform emulsion: (**a**) photograph of the separation of water-in-chloroform emulsion; (**b**) microscope photo and optical image of feed; (**c**) the corresponding filtrate; (**d**) the measured droplet size distribution of the feed; and (**e**) the corresponding filtrate.

To achieve oil/water phase separation, the aged porous brass was mounted onto a separation device with clamps and sealed with silicone gaskets (Figure 9a). Taking the Sudan III-dyed water-in-chloroform emulsion as a typical example, the feed emulsion was milky (inset in Figure 9b), and numerous dispersed droplets of size below 5.0 μm were distributed in the feed (Figure 9b). The filtrate became totally transparent (inset in Figure 9c), and there were no water droplets observed in the microscopic image (Figure 9c), which meant that the dispersed droplets were successfully removed. The corresponding dynamic light scattering (DLS) curve revealed that the water droplet diameters of the feed emulsion were in a wide range of 0.2–3.0 μm (Figure 9d), while only several strong peaks of less than 6.0 nm were observed for the filtrate (Figure 9e), further illustrating the effective separation for water-in-chloroform emulsion.

When the water-in-chloroform emulsion was introduced to touch the aged brass surface, oil was captured quickly while water was repelled completely. An oil film bridging process would rapidly occur among porous skeletons so that pores in the brass were readily filled by oil [58]. So, the oil film could block the large water droplets released by the demulsification from directly passing through the channels in the aged brass (Video S1). Meanwhile, the released small water droplets entered the tortuous pore channels under gravity and coalesced with each other by physical collision, forming large droplets that were easy to prevent [59,60,61]. Consequently, tortuous channels increased the permeation resistance of the water droplets and permitted more water droplets to coalesce in the porous brass, thus greatly enhancing the probability of demulsification.

**Figure 10:**
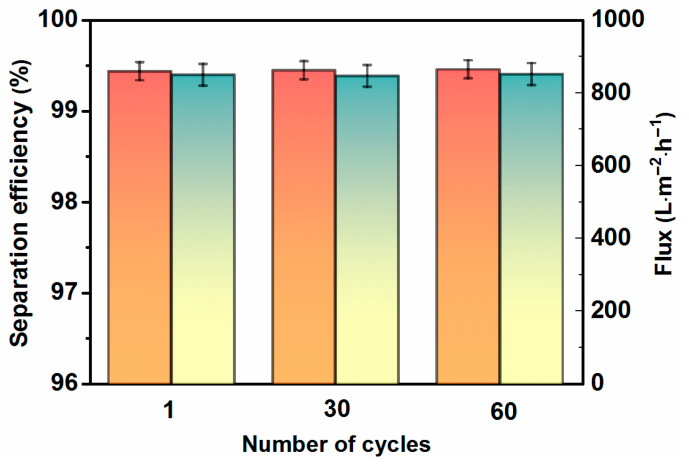
Changes in separation efficiency and permeation flux of water-in-chloroform emulsion over 60 cycles.

The service life of the aged brass was studied for practical applications. The aged brass exhibited excellent separation performance during filtration with a separation efficiency greater than 99.4% and a permeation flux of about 851 L·m^−2^·h^−1^ for water-in-chloroform emulsion with a height of 10 cm (Figure 10). Obviously, the separation efficiency of the aged brass was superior to that of some reported metal membranes [62,63]. The high efficiency can be attributed to its narrow range of tortuous channel size distribution (Figure 2h), which increases the efficiency of the separation process [64]. After each separation, the aged brass was reused by drying at room temperature. After recycling for 60 cycles, no change was found in the separation efficiency and flux for water-in-chloroform emulsion. Moreover, similar effective separations were also achieved for other surfactant-stabilized emulsions, such as water-in-kerosene and water-in-dichloroethane emulsions. This result clearly demonstrated that the aged brass possessed excellent separation performance for water-in-oil emulsions.

### 2.6. Mechanical, Thermal, and Chemical Stability

**Figure 11:**
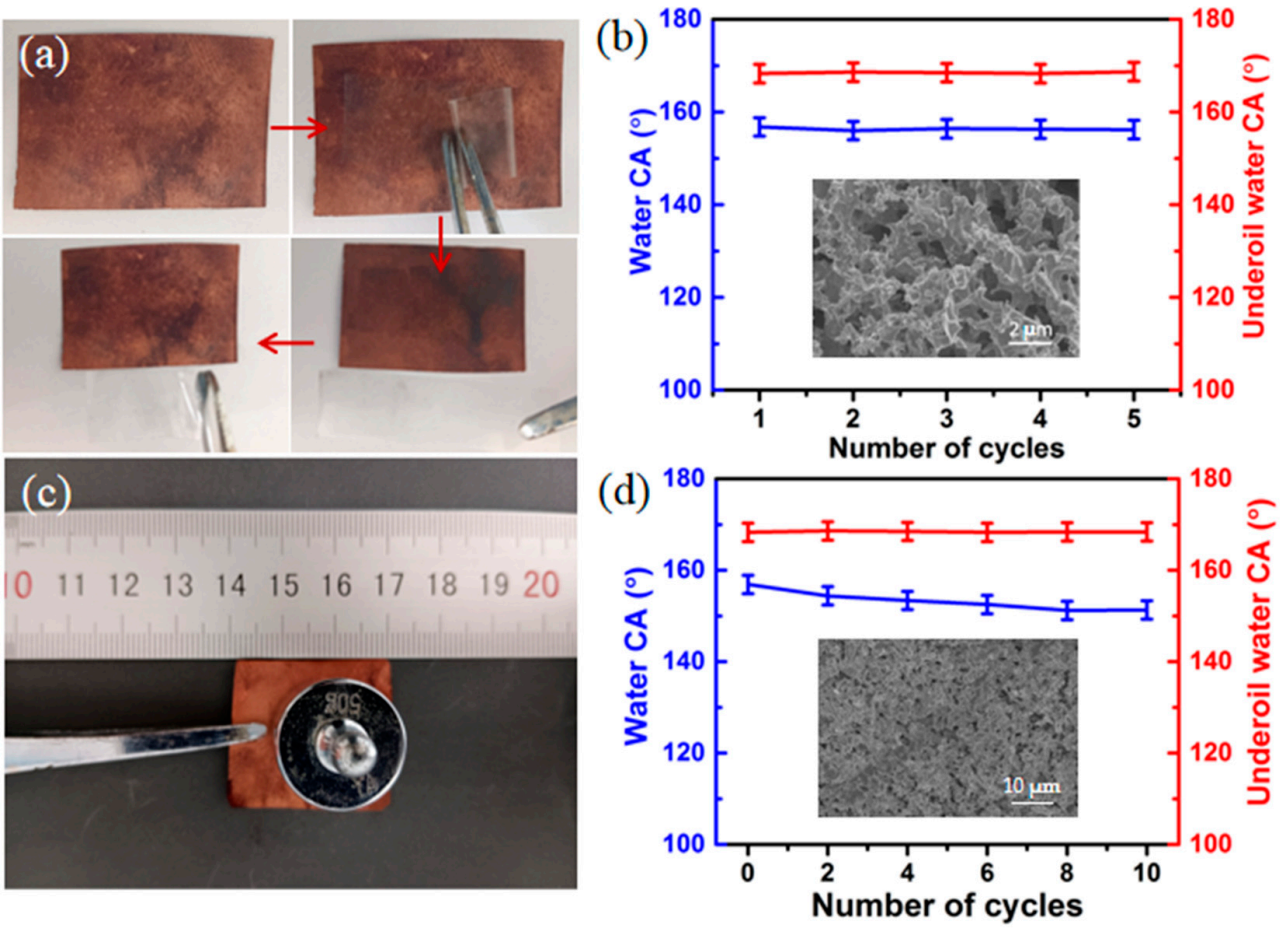
The mechanical stability of the aged brass in different working environments: (**a**) tape-peeling test; (**b**) variation of water CAs over tape-peeling cycles with the corresponding SEM image after 10 cycles in the inset; (**c**) sandpaper abrasion test; and (**d**) influence of abrasion cycles on the water CAs with the corresponding SEM image after 10 cycles in the inset.

Superhydrophobic surfaces are required to be stable in a harsh environment, such as mechanical friction, high temperature, or corrosive media. The tape-peeling test was performed on the aged brass surface using Scotch tape (No. 30403, Deli Group, Ningbo, China). In this test, the tape was pressed onto the aged brass, following which a roller of 200 g weight was rolled forward and then backward once. Only a very weak attachment was observed on the Scotch^®^ tape surface after 10 cycles (Figure 11a). However, the aged brass still had a water CA of 154°, and its underoil superhydrophobicity remained fairly stable (Figure 11b). Moreover, the morphological structure of the surface showed no obvious changes (inset in Figure 11b), indicating its good mechanical stability.

Then, an abrasion test was carried out by placing the aged sample face-down on a 1000-grit SiC sandpaper under a 50 g weight and dragging for a distance of 10 cm on the sandpaper, which was defined as one abrasion cycle. After eight abrasion cycles, the water CA slightly reduced from 156° to 151°, which could be attributed to the fact that rough sandpaper wore out the hierarchical structure on the aged surface. However, the exposed structure on the surface had the same phase as the substrate, and the exposed skeleton was gradually oxidized to Cu_2_O by O_2_ from the air (inset in Figure 11d). Further increase in grinding cycles did not show an obvious downward trend for static water CA. About 7 days after friction, the water CA of the sample again reached its maximum value and then remained constant. Meanwhile, the abraded brass could maintain underoil superhydrophobicity over 167° after 10 abrasion cycles.

**Figure 12:**
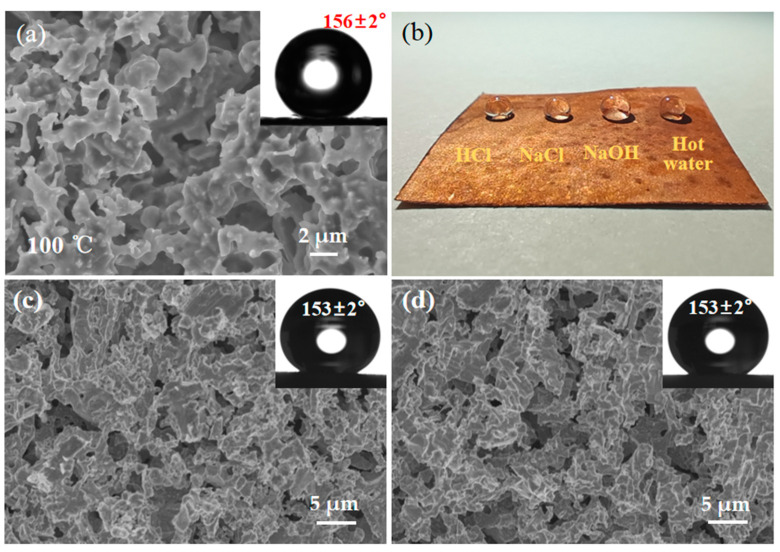
The thermal and chemical stability in different working environments: (**a**) the SEM images of the aged brass after heated at 100 °C with a corresponding water CA image in the inset; (**b**) the chemical stability of the aged brass against various corrosive drops; the SEM images of the aged brass after immersing into (**c**) 1 mM HCl solution; and (**d**) 1 mM NaOH solution for 10 h with corresponding water CA images in the inset.

Furthermore, the thermal stability was evaluated by heating the aged brass in the air at 100 °C and 150 °C for 12 h. After heating at 100 °C, the aged brass still maintained the water CA above 156° (inset in Figure 12a), and the original structure of the aged brass remained unchanged (Figure 12a). However, when the temperature increased to 150 °C, the water CA decreased to about 0 °C. This result indicates that the aged brass retains thermal stability at least up to 100 °C. Finally, the chemical stability of the aged brass was evaluated by corrosive media. As shown in Figure 12b, the water drops of 3% NaCl, 1 mM HCl, 1 mM NaOH, and 90 °C solution retained a spherical shape with CAs greater than 154°. Correspondingly, when the aged brass was immersed in kerosene, its CAs of salt, acid, base, and hot water still remained above 167°, demonstrating moderate chemical stability and corrosion resistance. The chemical durability of the aged surface was further tested by immersing the aged brass in weak acid (1 mM HCl solution) and alkali (1 mM NaOH solution) solutions for 10 h, respectively. When the aged brass reached the acid/base solution surface, a dense air protection layer could be formed on the surface of the aged brass [65]. The corrosion medium was difficult to approach and diffuse into the superhydrophobic material, resulting in the sample’s floating on the liquid surface steadily (Figure S5). So, the aged brass was forced into the corrosive solutions for 10 h, recording the water CAs after drying at room temperature. The water CA remained at about 153°, although there was a slight decrease observed after 10 h in both HCl and NaOH solutions (inset in Figure 12c,d). Compared with the aged brass, there is no apparent damage observed on the sample after the test (Figure 12c,d). This is attributed to the inherent chemical inertness of the Cu_2_O protective layer, delivering moderate chemical stability for the aged brass. However, the HCl solution would have a reaction with hydrophobic Cu_2_O as follows:

2HCl + Cu_2_O → CuCl_2_ + H_2_O (3)

After the Cu_2_O film was dissolved in an acidic solution, the fresh copper atoms were exposed and immediately reacted with oxygen dissolved in water, forming Cu_2_O on the aged brass surface. So, another reason why the surface of the aged brass could maintain superhydrophobicity might be that the dissolution rate of Cu_2_O was slower than the formation rate due to the low HCl concentration. This observation is basically consistent with the results reported in the previous literature [66,67]. Above all, the aged brass shows the potential practical application for oily sewage separation under harsh conditions.

## 3. Materials and Methods

### 3.1. Materials and Chemicals

Commercial brass foil (Cu_62_Zn_38_) with a thickness of 100 µm was purchased from Jona Hardware Company (Taizhou, China). Hydrochloric acid (HCl, 37%), petroleum ether, dichloromethane, n-hexane, and kerosene were bought from Sinopharm Chemical Reagent Co., Ltd. (Shanghai, China). Diesel was purchased from a local fueling station. Ethanol, xylene, and chloroform were obtained from Zhengzhou Junyi Chemical Reagent Co., Ltd. Sudan III (Zhengzhou, China) as dyed reagent was supplied by Shanghai Zhongqin Chemical Reagent Co., Ltd. (Shanghai, China). Span 80 was purchased from Aladdin Chemistry Co., Ltd. (Shanghai, China). All of the above agents were of analytical grade and used without further purification.

### 3.2. Superhydrophobic Porous Brass Fabricated by Dealloying and Aging

Briefly, porous brass was fabricated by dealloying and subsequent aging of pristine brass foil. Firstly, a commercially purchased brass foil (Cu62Zn38) was cut into slices with desired dimensions (denoted as pristine brass). The obtained brass was ultrasonically cleaned, in turn, by ethanol and distilled water to remove surface contamination. Then, dealloying was conducted by immersing the cleaned brass foil into a 37% HCl solution for 8 h at 80 °C to remove the Zn element. After dealloying, the reddish–brown sample was washed with water and ethanol (denoted as dealloyed brass). After being exposed to ambient air for 7 days, the aged brass could be used directly as separating material (denoted as aged brass).

### 3.3. Aging in Ambient Air and Organic Vapors

To study the mechanism behind the wettability transition, dried and undried dealloyed samples were exposed to four types of atmosphere, including ambient air, petroleum ether, dichloromethane, and n-hexane. For this purpose, eight freshly dealloyed samples were first rinsed thoroughly in distilled water, and then half of them were dried with cold air from a hair dryer. After that, these dried and undried samples were immediately stored in ambient air or vapors produced by 60 mL of organic compounds in closed glass bottles for 3 days. Every 24 h, these samples were taken out from each container to measure their water CAs. Following the measurement, these samples were immediately returned back to their containers.

### 3.4. Preparation and Separation of Water-in-Oil Emulsions

Different surfactant-stabilized water-in-oil emulsions were prepared by emulsifying 1.0 g of water and 0.4 g of Span 80 in 100 mL of oils, including chloroform, kerosene, and 1,2-dichloroethane under intensive stirring for 8 h. For emulsion separation, the aged brass was tightly fixed in the separation device with clamps and sealed with silicone gaskets. The as-prepared emulsion was poured into the device to carry out separation only driven by gravity. The oil phase would easily permeate through the brass, whereas water droplets could be effectively blocked. The water rejection efficiency of the aged brass was quantitatively calculated according to the following equation:

(4) $$R(\%)=(1-\frac{C_{f}}{Cp})\times 100\%$$

where *C*_f_ and *C*_p_ are the contents of water in the feed and filtrate, respectively. The permeate flux is determined by dividing the permeate volume by the product of the effective area and the penetration time under gravity with the height of feed emulsions of 10 cm according to the following equation:

(5) $$F=\frac{V}{St}$$

where *V* represents the permeate volume (L); *S* represents the effective area of the brass (m^2^), and *t* represents the penetration time (h), respectively.

### 3.5. Instruments and Characterization

All optical photographs were taken by a digital camera (Nikon D5000, Tokyo, Japan). The surface microstructure and element content of brass specimens were observed by a field emission scanning electron microscope (FESEM, Hitachi SU8000, Tokyo, Japan) equipped with an energy-dispersive X-ray spectrometer (EDS, AMETEK, Berwyn, PA, USA) attachment. The porosity and pore size distribution of the as-prepared brass were measured and analyzed by an automatic mercury porosimeter (Autopore V 9620, Micromeritics, Norcross, GA, USA). The specific surface area and average pore diameter distribution were analyzed by nitrogen adsorption/desorption isotherms using an automated adsorption apparatus (Autosorb-iQ-MP-C, Quantachrome, Boynton Beach, FL, USA). Attenuated total reflection Fourier-transform infrared spectra (ATR-FTIR) of the samples were recorded on a Brucker Tensor-37 spectrometer (Brucker Tensor, Billerica, MA, USA) equipped with a MIRacle ATR accessory (Pike Technologies, Fitchburg, WI, USA). The crystallinity and phase of the specimens were determined by X-ray diffractometer (XRD, Rikagu SmartLab system, Tokyo, Japan) using a Cu Kα radiation source. The chemical composition of the surface was identified by using a Laser Microscopic Confocal Raman Spectrometer (Senterra, Bruker, Billerica, MA, USA) at a laser wavelength of 532 nm. The surface chemical composition and oxidation state of the as-prepared samples were analyzed by X-ray photoelectron spectroscopy (XPS, Thermo Scientific ESCALAB 250Xi, Waltham, MA, USA), equipped with a monochromatic X-ray source (Al Ka, 1486.6 eV). The XPS spectra were fitted to a Shirley–Linear background with XPS peak 4.1 software. The average contact angle (CA) and sliding angle (SA) were calculated by measuring three different positions of the same sample with a 5 uL droplet by an Attension Theta system (PD-200, Biolin Scientific, Västra Frölunda, Sweden). The dispersion of the feed emulsions and the corresponding filtrates was obtained by taking optical images using an optical microscope (Leica DM4000, Wetzlar, Germany). The droplet diameter distribution in the emulsions and filtrates was calculated by dynamic light scattering (DLS) with Zetasizer Nano ZS (Malvern 3600, Malvern, UK). The water content in the filtrate was accurately determined by Karl Fischer Titrator (MKS-500).

## 4. Conclusions

A uniform microporous structure with an average pore size of 1.6 μm was produced by a simple chemical dealloying process of CuZn alloy foil. After dealloying, the sample exhibited superhydrophilic behavior but gradually became superhydrophobic over time, with the maximum water CA being achieved after 7 days. This is because the surface of the dealloyed brass was oxidized to Cu_2_O by O_2_ from the air to induce wettability conversion. Such a superhydrophobic surface showed excellent long-term storage durability for storing the sample in an ambient atmosphere. By combining superhydrophobicity with small pore channels, the novel porous brass could be employed to separate water-in-oil emulsions with high efficiency and repeatability. Thanks to the hierarchical structures on the surface having the same phase as the substrate, the superhydrophobic porous brass demonstrated good mechanical, thermal, and chemical stability. This simple fabrication technique may provide a new avenue for the design and fabrication of porous materials used in oil/water separation.

## Data Availability

Our research data will be available on demand.

## Supplementary Materials

The following supporting information can be downloaded at https://www.mdpi.com/article/10.3390/molecules28186509/s1, Figure S1: EDS spectra analysis and mapping images: (a) pristine brass and (b) dealloyed brass; Figure S2: CuLMM Auger transitions of pristine brass, dealloyed brass, and aged brass; Figure S3: The kerosene CA of the aged brass surface in air; Figure S4: High-resolution C1s spectra of (a) the dealloyed brass and (b) the aged brass; Figure S5: The aged brass floated on the liquid surface of 1 mM HCl solution: Video S1: Separation process of surfactant-stabilized water-in-chloroform emulsion.

## Sample Availability

Not available.
