# Supplementary material for: Fabrication of Superhydrophobic Porous Brass by Chemical Dealloying for Efficient Emulsion Separation
Source: Molecules. 2023 Sep 8;28(18):6509. doi: 10.3390/molecules28186509 (PMC10534338; doi:10.3390/molecules28186509)
Supplement: Supplementary file 1 [file molecules-28-06509-s001.zip › supplementary materials.pdf]

Supporting Information

Fabrication of Superhydrophobic Porous Brass by Chemical Dealloying for Efficient Emulsion Separation

Yanbiao Zhou, Qingqing Ye, Yongjun Han *, Guoxu He and Changdong Chen *

School of Chemistry and Environmental Engineering, Pingdingshan University, Pingdingshan 467000, China

* Correspondence: hyj@pdsu.edu.cn (Y.H.); 2749@pdsu.edu.cn (C.C.)

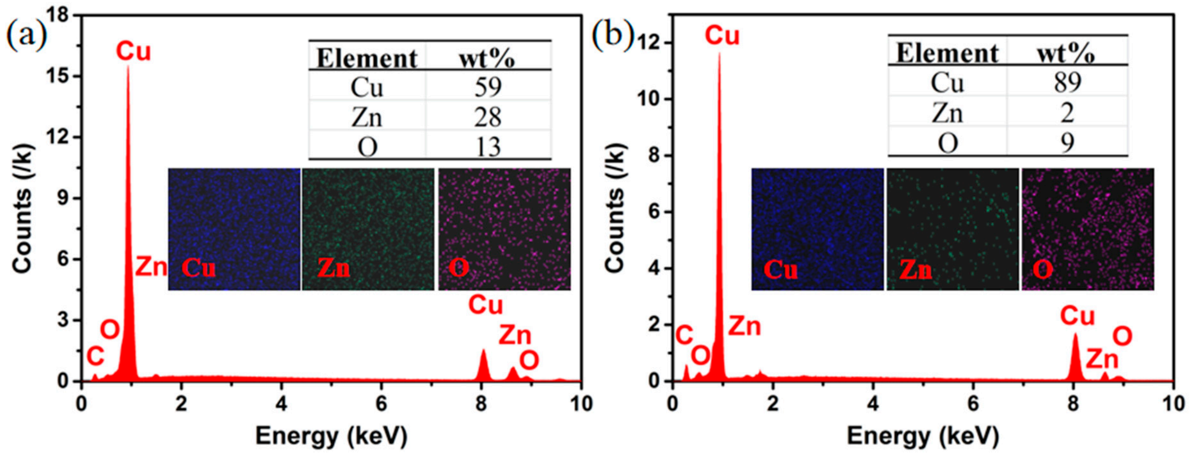

Figure S1. EDS spectra analysis and mapping images: (**a**) pristine brass and (**b**) dealloyed brass.

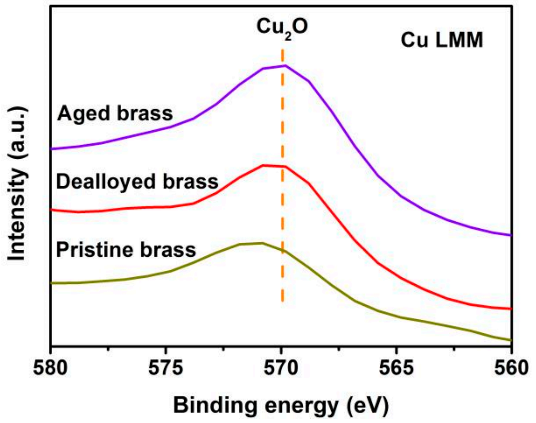

Figure S2. Cu_{LMM} Auger transitions of pristine brass, dealloyed brass and aged brass.

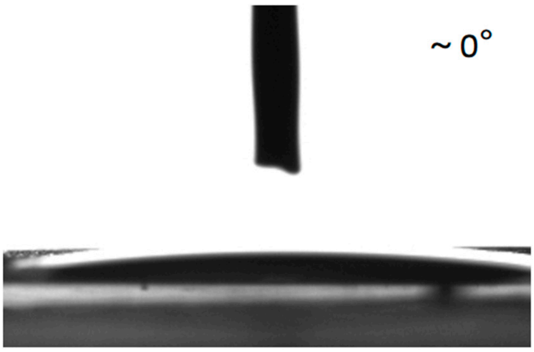

Figure S3. The kerosene CA of the aged brass surface in air.

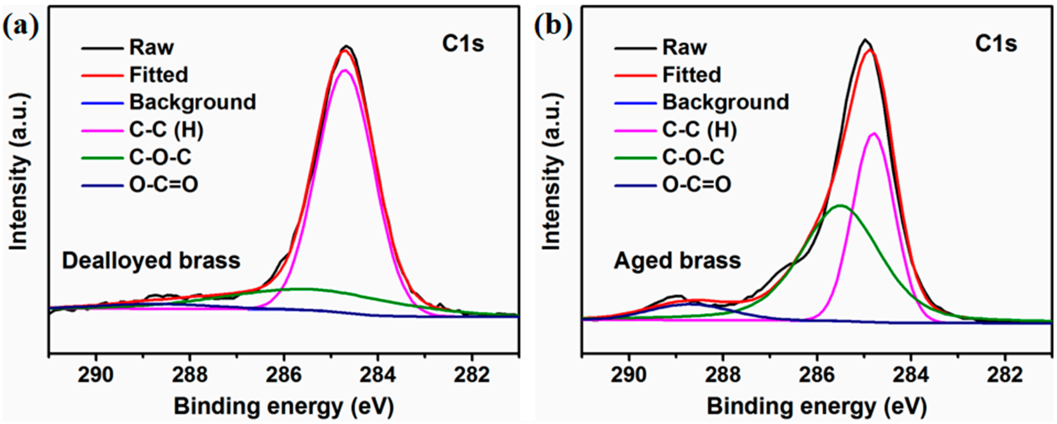

Figure S4. High resolution C1s spectra of (**a**) the dealloyed brass and (**b**) the aged brass.

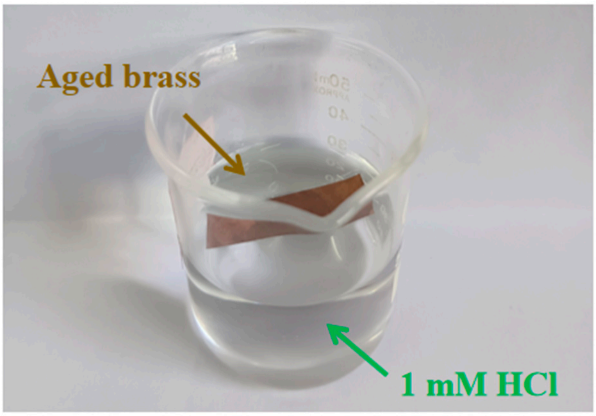

Figure S5. The aged brass floated on the liquid surface of 1 mM HCl solution.
